# Complete Genome Sequence of the Amino Acid-Fermenting *Clostridium propionicum* X2 (DSM 1682)

**DOI:** 10.1128/genomeA.00294-16

**Published:** 2016-04-14

**Authors:** Anja Poehlein, Katja Schlien, Nilanjan Pal Chowdhury, Gerhard Gottschalk, Wolfgang Buckel, Rolf Daniel

**Affiliations:** a Genomic and Applied Microbiology and Göttingen Genomics Laboratory, Georg-August University Göttingen, Göttingen, Germany; b Laboratorium für Mikrobiologie, Fachbereich Biologie, Philipps-Universität, Marburg, Germany; c Max-Planck-Institut für terrestrische Mikrobiologie, Marburg, Germany

## Abstract

*Clostridium propionicum* is a strict anaerobic, Gram positive, rod-shaped bacterium that belongs to the clostridial cluster XIVb. The genome consists of one replicon (3.1 Mb) and harbors 2,936 predicted protein-encoding genes. The genome encodes all enzymes required for fermentation of the amino acids α-alanine, β-alanine, serine, threonine, and methionine.

## GENOME ANNOUNCEMENT

The strictly anaerobic, Gram positive, and rod-shaped bacterium *C. propionicum* belongs to the cluster XIVb of nonpathogenic clostridia (1). *C. propionicum* is able to produce propionate, acetate, ammonia, and CO_2_ by fermentation of α-alanine, β-alanine, or serine (2) via the nonrandomizing acryloyl-CoA pathway (3). This organism was originally isolated in 1946 from black mud of the San Francisco Bay (USA) by Cardon and Barker (2).

The MasterPure complete DNA purification kit (Epicentre, Madison, USA) was used to isolate chromosomal DNA of *C. propionicum* X2 (DSM 1682). Sequencing was done by a combined approach using the 454 GS-FLX pyrosequencing system (Roche Life Science, Mannheim, Germany) and the HighSeq 2000 system (Illumina, San Diego, CA, USA). Shotgun sequencing libraries were prepared according to protocols of the manufacturers. Sequencing resulted in 45,315,878 Illumina reads (2 × 100 bp paired end) and 218,691 pyrosequencing reads. The *de novo* hybrid assembly was performed with the Roche Newbler assembly and Mira 3.4 software (4) by employing 3,000,000 randomly selected Illumina reads and all 454 pyrosequencing reads. The average coverage was 24.17 (454) and 92.83 bp (Illumina). Gap closure was performed by PCR-based approaches, Sanger sequencing of the PCR products, and employing the Gap4 (v4.11) software of the Staden package (5). The complete genome of *C. propionicum* X2 (DSM 1682) comprises one circular chromosome (3.1 Mb) with an overall G+C content of 44.08 %. Automatic gene prediction was performed using the software Prodigal (6). Identification of rRNA and tRNA genes was done with RNAmmer (7) and tRNAscan (8), respectively. The IMG/ER (Integrated Microbial Genomes/Expert Review) system (9) was used for automatic annotation, which was subsequently manually curated by using the Swiss-Prot, TREMBL, and InterPro databases (10). We could identify 7 rRNA operons, 61 tRNA genes, 2,060 protein-encoding genes with function prediction, 876 genes coding for hypothetical proteins, and 9 pseudogenes. The genome harbors all genes encoding enzymes necessary for the fermentation of α-alanine, β-alanine, serine, threonine, and methionine. We identified three identical copies of gene cluster *acrABC*, which encodes the acrylyl-CoA reductase and the two subunits of the electron transfer flavoprotein. This enzyme complex catalyzes the NADH-dependent reduction of acrylyl-CoA to propionyl-CoA (11) during fermentation of alanine. Other enzymes involved in this pathway such as propionate CoA transferase (Pct), lactyl-CoA dehydratase (LcdCAB) were also present. Identical gene clusters were located in the recently published genome of *C. neopropionicum* (12). The genome of *C. propionicum* harbors a complete *rnf* cluster (*rnfABCDEG*) and a gene encoding *Re*-citrate synthase (13) upstream of the gene coding for aconitase. Genes encoding proteins necessary for chemotaxis are also present and flanked by two large clusters encoding proteins for flagellar biosynthesis, and a third cluster is located elsewhere in the genome.

### Nucleotide sequence accession number.

The genome sequence has been deposited in GenBank under accession number CP014223.
